# Supplementary material for: Effects of Bismuth Exposure on the Human Kidney—A Systematic Review
Source: Antibiotics (Basel). 2022 Dec 2;11(12):1741. doi: 10.3390/antibiotics11121741 (PMC9774474; doi:10.3390/antibiotics11121741)
Supplement: Supplementary file 1 [file antibiotics-11-01741-s001.zip › antibiotics-2035684-supplementary.pdf]

Effects of bismuth on the human kidney – a systematic review

Supplementary file 1

Excluded articles during the full-text screening, with reasons:

- Kidney implications were reported, but not related with bismuth [76,80–83]
- Treatment using bismuth, but no effects, conditions, or diseases were reported
    - Simple renal cyst [84]
    - *Helicobacter pylori* infection on long-term risk of peptic ulcer bleeding [85,86]
- Included bismuth accumulation without discussing renal effects [87,88]
- Review study:
    - nephrological aspects of peptic ulcer therapy considering adverse effects on the kidneys [89]
    - discussing drug-drug interaction of proton pump inhibitors including bismuth, but no renal effect was tested [90]
    - *Helicobacter pylori* infection [91]
- Comments about a previous study [92]
- Animal study not stating this study category in the title nor in the abstract [93–95].
- Cell culture study [96]
- Conference abstract [97]

Reference list of the excluded articles

80. Smith DMJ, Pitcock JA, Murphy WM. Aluminum-containing dense deposits of the glomerular basement membrane: identification by energy dispersive X-ray analysis. Am J Clin Pathol. 1982;77(3):341-346. doi:10.1093/ajcp/77.3.341

81. McCague A, Joe VC. A Case of Argyria and Acute Leukopenia Associated with the Use of an Antimicrobial Soft Silicone Foam Dressing. J Burn Care Res. 2016;37(5):e493-6. doi:10.1097/BCR.0000000000000275

82. de Vroom SL, van Hest RM, van Daalen F V, et al. Pharmacokinetic/pharmacodynamic target attainment of ciprofloxacin in adult patients on general wards with adequate and impaired renal function. Int J Antimicrob Agents. 2020;56(5):106166. doi:10.1016/j.ijantimicag.2020.106166

83. Geenen L, Nonnekens J, Konijnenberg M, Baatout S, De Jong M, Aerts A. Overcoming nephrotoxicity in peptide receptor radionuclide therapy using [177Lu]Lu-DOTA-TATE for the treatment of neuroendocrine tumours. Nucl Med Biol. 2021;102-103:1-11. doi:10.1016/j.nucmedbio.2021.06.006

84. Holmberg G, Hietala SO. Treatment of simple renal cysts by percutaneous puncture and instillation of bismuth-phosphate. Scand J Urol Nephrol. 1989;23(3):207-212. doi:10.3109/00365598909180843

85. Chan FKL, Ching JYL, Suen BY, Tse YK, Wu JCY, Sung JJY. Effects of Helicobacter pylori infection on long-term risk of peptic ulcer bleeding in low-dose aspirin users. Gastroenterology. 2013;144(3):528-535. doi:10.1053/j.gastro.2012.12.038

86. Hsu C-H, Hu H-Y, Huang N, Chang S-S. Early eradication has a lower risk of peptic ulcer bleeding in Helicobacter pylori-infected chronic kidney disease patients. Eur J Intern Med. 2016;33:112-117. doi:10.1016/j.ejim.2016.06.035

87. Gatti AM, Bosco P, Rivasi F, et al. Heavy metals nanoparticles in fetal kidney and liver tissues. Front Biosci - Elit. 2011;3 E(1):221-226. doi:10.2741/e236

88. Kratochwil C, Schmidt K, Afshar-Oromieh A, et al. Targeted alpha therapy of mCRPC: Dosimetry estimate of 213Bismuth-PSMA-617. Eur J Nucl Med Mol Imaging. 2018;45(1):31-37. doi:10.1007/s00259-017-3817-y

89. Burgess E, Muruve D. Renal effects of peptic ulcer therapy. Drug Saf. 1992;7(4):282-291. doi:10.2165/00002018-199207040-00004

90. Ogawa R, Echizen H. Drug-drug interaction profiles of proton pump inhibitors. Clin Pharmacokinet. 2010;49(8):509-533. doi:10.2165/11531320-000000000-00000

91. Zhong M-F, Li J, Liu X-L, Gong P, Zhang X-T. TCM-Based Therapy as a Rescue Therapy for Re-Eradication of Helicobacter pylori Infection: A Systematic Review and Meta-Analysis. Evidence-based Complement Altern Med. 2022;2022. doi:10.1155/2022/5626235

92. Weil J, Bell GD, Powell K. Disposition of bismuth and renal function. Aliment Pharmacol Ther. 1992;6(3):395-397.

93. Sun H, Li H, Harvey I, Sadler PJ. Interactions of bismuth complexes with metallothionein(II). J Biol Chem. 1999;274(41):29094-29101. doi:10.1074/jbc.274.41.29094

94. Li L, Lu Y, Lin Z, et al. Ultralong tumor retention of theranostic nanoparticles with short peptide-enabled active tumor homing. Mater horizons. 2019;6(9):1845-1853. doi:10.1039/C9MH00014C

95. Stacy A, Andrade-Oliveira V, McCulloch JA, et al. Infection trains the host for microbiota-enhanced resistance to pathogens. Cell. 2021;184(3):615-627.e17. doi:10.1016/j.cell.2020.12.011

96. Liu Y, Zhuang J, Zhang X, et al. Autophagy associated cytotoxicity and cellular uptake mechanisms of bismuth nanoparticles in human kidney cells. Toxicol Lett. 2017;275:39-48. doi:10.1016/j.toxlet.2017.04.014

97. Goldberg D, Parker V, King M. Intentional overdose in a teenager with late salicylate toxicity secondary to bezoar formation. Crit Care Med. 2014;42(12):A1651. doi:10.1097/01.ccm.0000458713.75100.94
